# Rapid and Sensitive Detection of Hepatitis C Virus in Clinical Blood Samples Using Reverse Transcriptase Polymerase Spiral Reaction

**DOI:** 10.4014/jmb.1910.10041

**Published:** 2019-12-24

**Authors:** Wenying Sun, Ying Du, Xingku Li, Bo Du

**Affiliations:** 1 Clinical Laboratory, the Second Affiliated Hospital of Harbin Medical University, Harbin, 50086, P.R. China; 2 Department of Experimental Diagnosis, Heilongjiang Provincial Hospital, Harbin, 150036, P.R. China; 3 Experimental Research Center, the Second Affiliated Hospital of Harbin Medical University, Harbin, 150086, P.R. China

**Keywords:** Hepatitis C virus, polymerase spiral reaction, rapid detection, clinical detection

## Abstract

This study established a new polymerase spiral reaction (PSR) that combines with reverse transcription reactions for HCV detection targeting 5’UTR gene. To avoid cross-contamination of aerosols, an isothermal amplification tube (IAT), as a separate containment control, was used to judge the result. After optimizing the RT-PSR reaction system, its effectiveness and specificity were tested against 15 different virus strains which included 8 that were HCV positive and 7 as non-HCV controls. The results showed that the RT-PSR assay effectively detected all 8 HCV strains, and no false positives were found among the 7 non-HCV strains. The detection limit of our RT-PSR assay is comparable to the real-time RT-PCR, but is more sensitive than the RT-LAMP. The established RT-PSR assay was further evaluated for detection of HCV in clinical blood samples, and the resulting 80.25% detection rate demonstrated better or similar effectiveness compared to the RT-LAMP (79.63%) and real-time RT-PCR (80.25%). Overall, the results showed that the RT-PSR assay offers high specificity and sensitivity for HCV detection with great potential for screening HCV in clinical blood samples.

## Introduction

Hepatitis C is a progressive liver disease caused by the hepatitis C virus (HCV), which is a single-stranded RNA virus belonging to the *Hepacivirus* genus of the *Flaviviridae* family [1, 2]. According to a World Health Organization (WHO) report, HCV infection is still one of the major public health problems worldwide, especially in Asia [3, 4]. In China, the HCV-infected population has been estimated to be 14 million (with prevalence of 1.0%), with the genotype 1b accounting for 70% of four HCV genotypes (1, 2, 3 and 6) that have been reported to date [5, 6]. Unsafe injection practices, drug diversion and unsafe blood transfusions have led to a continuing increase in morbidity and mortality [7]. Therefore, the establishment of a reliable and accurate method for identification of HCV infection in clinics as well as in large-scale screening of healthy populations is of great significance for patient management and epidemic study.

During the past decades, a number of diagnostic methods have been developed to detect HCV infection, including enzyme immunosorbent assays (EIA), recombinant immunoblot assays (RIBA), real-time PCR assay and isothermal amplification technologies (IATs), etc. [8-10]. Serological screening using anti-HCV antibodies has been used as the “gold standard” to diagnose HCV infection, but this method incidentally leads to false negative results especially in the early stages of HCV infections [11, 12]. Molecular techniques such as real-time PCR can detect HCV infection much earlier in the infection process, however, the assay requires high-precision, expensive equipment and skilled technicians [7] which largely limits it use.

**Fig. 1:**
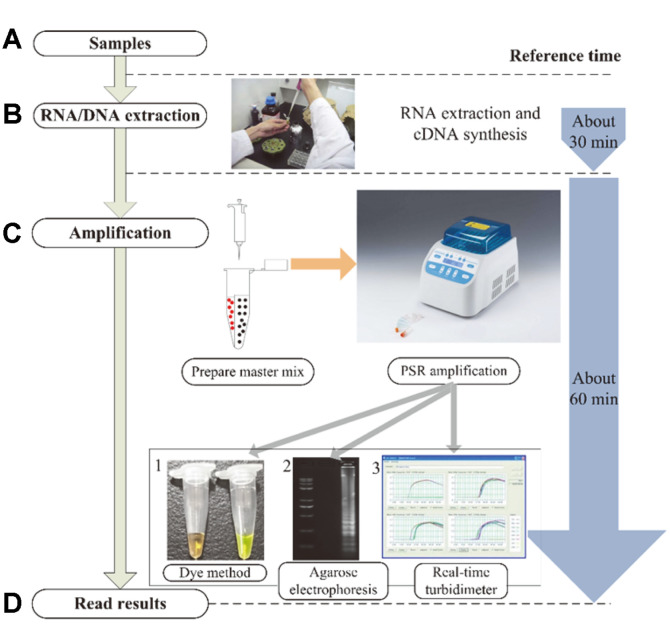
Schematic diagram of RT-PSR concept. (**A**) Collection of samples. (**B**) Sample pretreatment. (**C**) Sample loading and incubation. (**D**) Results detection. 1-3: the results of dye method, agarose amplification and real-time turbidity detection, respectively.

Ideally, a diagnostic method capable of providing rapid, sensitive, affordable, equipment-free and easy judgement was considered to be compatible with developing countries and economically underdeveloped areas. In recent years, isothermal nucleic acid amplification techniques (INAATs) have steadily emerged to provide an analytical solution that overcomes some disadvantages associated with traditional methods [13-15]. In most of INAATs assays, loop-mediated isothermal amplification (LAMP) has been extensively established and applied for the rapid detection of various bacterial, viral, fungal and parasitic infections [16, 17]. Since the first RT-LAMP for HCV detection, using the strand displacement activity of Bst DNA polymerases, many new RT-LAMP assays have been developed, but the risk of contamination and false positive results are still troublesome factors with LAMP [12]. Recently, the polymerase spiral reaction (PSR) method, employing a new isothermal amplification technology, has been conceptualized and developed to alleviate several of the shortcomings of previously developed INAAT methods [18, 19]. The technique only needs two primers, which are designed to contain two unrelated exogenous sequences (forward/reverse sequences) at each of 5’ end and the remaining sequence corresponding to the subject target region [20]. The PSR procedure can be completed under isothermal conditions within 1 h without complicated equipment and the products can be visualized after addition of any fluorescent dye such as SYBR Green I or hydroxyl-naphthol blue (HNB) (Fig. 1). Furthermore, compared to other existing assays, PSR does not require an initial pre-denaturation and provides advantages like rapidity, efficiently and sensitivity, giving it potential application value in field diagnosis [21, 22].

In the present study, we designed and evaluated an isothermal amplification technology (RT-PSR) by targeting the HCV 5’UTR conserved region, which is considered to be the gold standard for genotyping and is highly conserved in all HCV strains [23]. The extracted HCV-RNA template was reverse transcribed and the cDNA was amplified for RT-PSR amplification. Our study demonstrated that, compared to RT-LAMP and RT-PCR, the RT-PSR is a fast, effective and sensitive assay for the detection of HCV and has great potential for clinical diagnosis and the epidemic study of chronic hepatitis C.

## Materials and Methods

### Blood Sample Collection and cDNA Preparation

A total 162 in-patients who have suspected chronic hepatitis C infection were randomly selected from the Second Affiliated Hospital of Harbin Medical University (China). Patient information such as age, sex and serological screening results for HCV was recorded. As controls, 18 healthy volunteers were selected from among the hospital employees. All volunteer donors signed an informed consent form and the research was also approved by the Institutional Review Board and Ethics Committee of the Harbin Medical Sciences University. Blood samples were collected from patients and controls. Total RNAs were extracted from 0.2 ml of whole blood using a commercial extraction kit (RNB100-GenElute Total RNA Purification Kit, Merck KGaA, Germany) by following the manufacturer’s instructions. The RNAs were subjected to a reverse transcription process to generate cDNA as instructed by the TaqMan MicroRNA Reverse Transcription Kit (Thermo Fisher Scientific, China). The cDNA products were subsequently separated and purified by a 2.0% agarose gel with a DNA gel extraction kit (TIANGEN, China), and then stored at -20°C for further use.

To better evaluate the effectiveness and specificity of RT-PSR for HCV detection, the core regions of HCV-positive DNAs from the published standard strains of HCV-HM674631 (1a), HCV-HEBEI L02836 (1b), HCV-AF034629 (2a), HCV-HK10U10197 (3a), HCV-STD11443 (3b), HCV-HK2U10198 (6a) were synthesized and cloned into the pAR2 expression vector. The plasmid DNAs extracted from the expression vectors were tested by RT-PSR to determine specificity and effectiveness. In addition, DNA extracted from 7 non-HCV strains was used as negative controls. The results were compared to RT-LAMP and RT-PCR (Table 1).

### Primers for RT-PSR, RT-LAMP and Real-Time RT-PCR

**Fig. 2:**
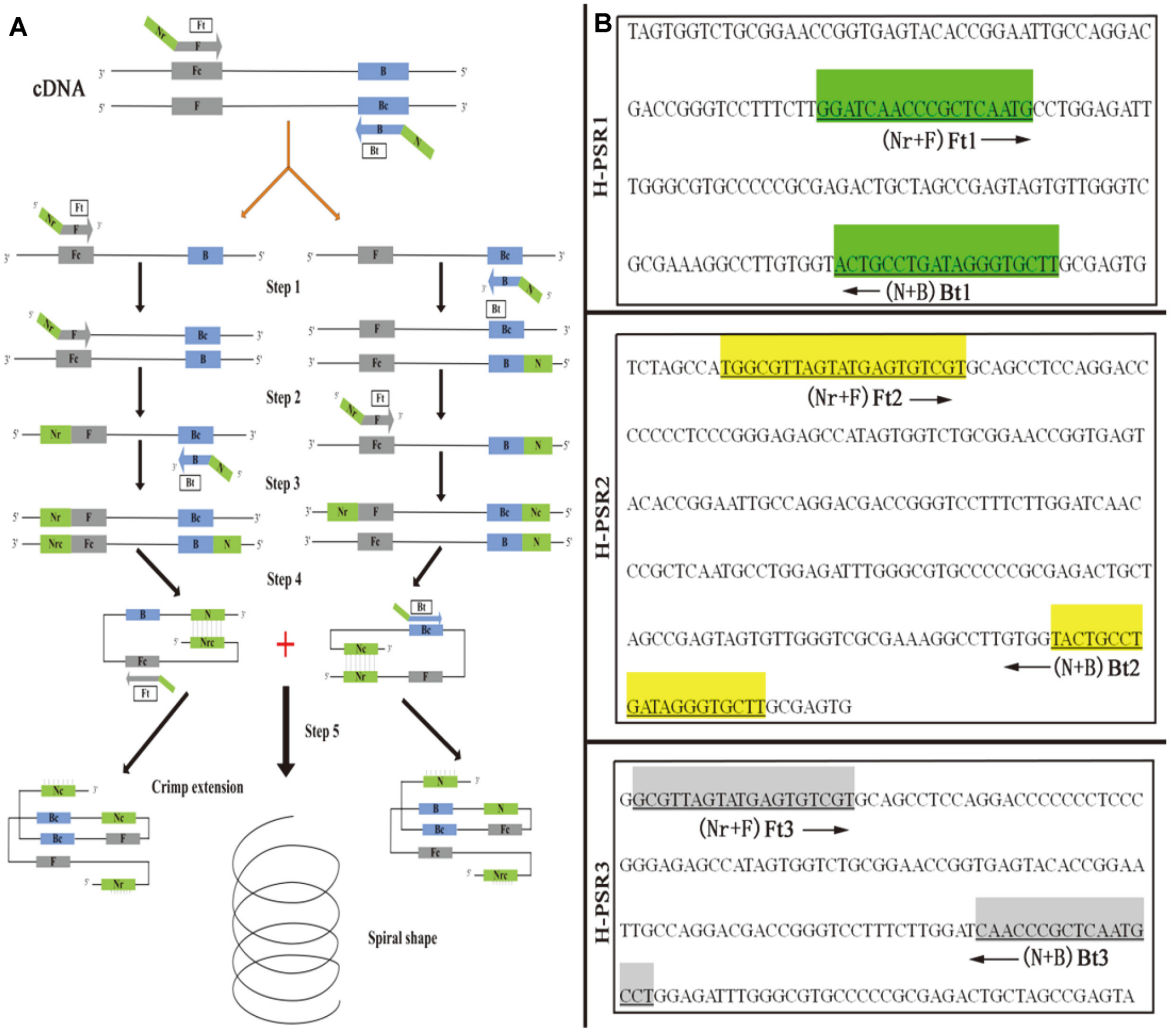
Schematic description of primer targeting of RT-PSR assay. (**A**) Schematic diagram of RT-PSR assay. (**B**) Binding region of three RT-PSR specific primer pairs. Arrows indicate the direction of amplification extension.

**Fig. 3:**
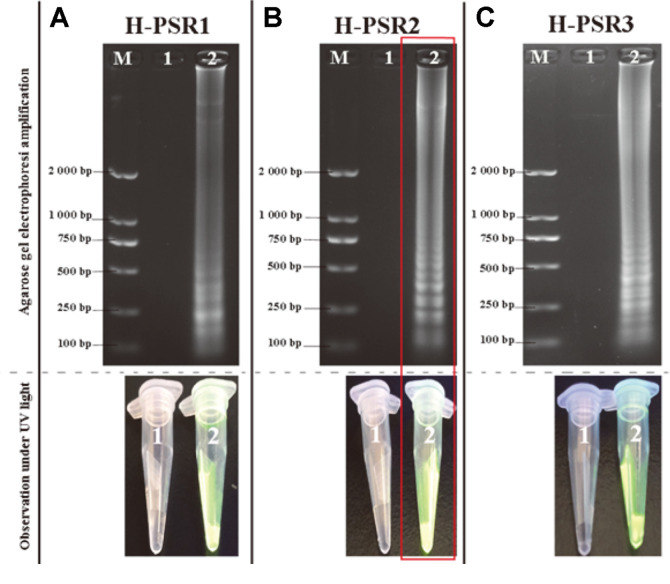
Amplification results for RT-PSR assay by three pairs of specific primers. (**A-C**) Amplified DNA bands shown under UV light after separation by agarose gel electrophoresis for H-PSR1, H-PSR2 and H-PSR3, respectively; M: Standard DNA marker; 1. negative control; 2. detection of HCV reference strain. (The primer combinations with the highest amplification efficiency among the three pairs were marked in red.)

To obtain an optimized test result, three pairs of HCV-specific primers were selected by targeting the 5’ untranslated region, which has been demonstrated to be highly conserved according to analysis from the GenBank database (Accession No: GQ418245), by using Primer Premier 5.0 (PREMIER Biosoft International Co., USA). The schematic diagram of PSR amplification and primer-corresponding binding regions are shown in Fig. 2, and the sequences of these primers are shown in Table 2. The specificity of the primers was checked by Primer-BLAST analysis of the NCBI sequence database to avoid non-specific reactions. Primers used for the RT-LAMP assay of HCV were synthesized as described by Kargar *et al*. [13], and consist of a set of six primers having two inner primers (Figs. 3 and B3), two outer primers (FIP and BIP) and two loop primers (LF and LB) recognizing eight distinct sequences of the conserved target region. All the oligonucleotide primers were synthesized by Genewiz Biotech Co., Ltd. (China) and listed in Table 2. A pair of real-time RT-PCR primers (BK1: CGGGAGAGCCATAGTGGT/ BK2: CAAGCACCCTATCAGGCA) and a TaqMan probe (CAAGGCCTTTCGCGACCCAA) was provided by Khalvati *et al* [24].

### System Optimization of RT-PSR

The polymerase spiral reaction was carried out at 60°C for 1 h and the reaction mixture contained the following components: 3 µl of 10 × ThermolPol reaction buffer, 1.5 µl (12 U) of Bst DNA polymerase large fragment (New England Biolabs, USA), 3.0 µl (0.8 M) betaine (Sigma-Aldrich, USA), 3 µl (4 mM) MgSO_4_ (Sigma-Aldrich), 2.5 µl (1.0 mM) dNTPs (Takara Bio, China), 2 µl of an appropriate concentration of target cDNA (Hebei L02836 used for positive control and sterilized deionized water used for blank control) and 1.6 µM primers (Ft and Bt) of each pair (H-PSR1, H-PSR2, H-PSR3). The total reaction volume was adjusted to 30 µl with sterilized deionized water. For rapid, clear and simple acquisition of results, an isothermal amplification tube (IAT, Guangzhou Hua-feng Biological Co., Ltd., China) was introduced and the SYBR Green I (2000 ×) dye (Solarbio, China) was added on one side and reaction mixture was added on the other side before the reaction. The positive reaction of the amplification was visualized under ultraviolet light as shown by green fluorescence, and the orange color was considered as negative. Meanwhile, 5 µl of amplification products was further analyzed by electrophoresis on 1.8% agarose gel and photographed. The conditions of amplification were optimized by gradient PSR under temperatures between 61°C and 71°C. The conditions for the reaction were optimized by testing with the concentrations of MgSO_4_ from1.0 mM to 8.0 mM, *Bst*-DNA polymerase from 6 U to 18 U, dNTP from 1.0 mM to 4.0 mM, and the concentration of betaine from 0.6 M-1.4 M. All the optimized test results were recorded by using a real-time turbidimeter (EIKEN Chemical Co., Ltd., Japan).

### RT-LAMP Assay

The RT-LAMP assay was performed at 63.5°C for 60 min as recommended by Kargar *et al*. The isothermal amplification tubes were tested at corresponding conditions and sterilized deionized water was set as blank control (repeated ≥3 times).

### Real-Time PCR

Real-time RT-PCR was performed by using the Bio-Rad iQ5 system (Bio-Rad, USA) following the manufacturer’s instructions for the specific reaction systems: an initial denaturation at 95°C for 3 min followed by 40 cycles of 95°C for 15 sec and 59°C for 1 min. Fluorescent measurements were recorded and analyzed during each extension step and amplification plots were automatically generated by the system at the end of each reaction. For each reaction, 2 µl of cDNA template, 500 nM of BK1/BK2, 400 nM TaqMan probe was added to 20 µl of SYBR Green I master mix (Thermo Fisher, China) and the amplification reactions were performed in triplicate.

## Results

### Validation and Optimization of RT-PSR Assay

The mechanism of the PSR method is illustrated in Fig. 2, where we take a single strand in the target sequence as an example (the amplification mechanism of another single strand is the same). When a suitable temperature is reached (60~65oC), the existence of betaine will turn the cDNA into a dynamic equilibrium of single and double strands. The specific primer Ft can specifically recognize the Fc segment on the target sequence and extend to the 3‘ end under the action of BST DNA polymerase, forming the DNA double strand structure in step 1. Subsequently, the double strand was broken down into a free DNA single strand (step 2) under the action of betaine. The B segment of another primer Bt combined with the B segment of one single strand and extended to 3’ (Step 3). The resulting double chain breaks again (step 4), and the N segment rotates and combines with the Nrc segment to form a spiral structure. Finally, the 3’ end of Nrc continues to extend to form a longer spiral structure (step 5).

The success of RT-PSR amplification depends on the specificity of the designed primers, but also can be impacted by other components in the system. In order to select the optimal reaction system, we successively optimized the conditions by changing reaction conditions such as the concentrations of dNTPs, Mg^2+^, betaine, *Bst*-DNA polymerase, and conditions of amplification such as temperature and incubation time. The reaction results were recorded with a real-time turbidimeter and the detailed optimization process is shown in Table 3. The final optimized conditions of RT-PSR were incubation for 45 min at 63oC, 1.5 mM dNTPs, 4 mM Mg^2+^, 1.2 M betaine, and 12 U/tube of Bst DNA polymerase.

In this study, three sets of RT-PSR specific primers targeting the 5’ UTR highly conserved region sequences were tested. The results showed that all three pairs of primers successfully amplified positive cDNA at the predicted size, whereas no bands were found in negative controls. Among them, the combination of H-PSR2 primers showed the best amplification effects under conventional system incubation. Mixing the chromogenic substrate SYBR Green I on one side carefully with the amplification products on the other side after 1 h of incubation, the positive amplification products exhibited bright green fluorescence, while the negative sample remained orange (Fig. 3).

### Effectiveness and Specificity of RT-PSR Assay

**Fig. 4:**
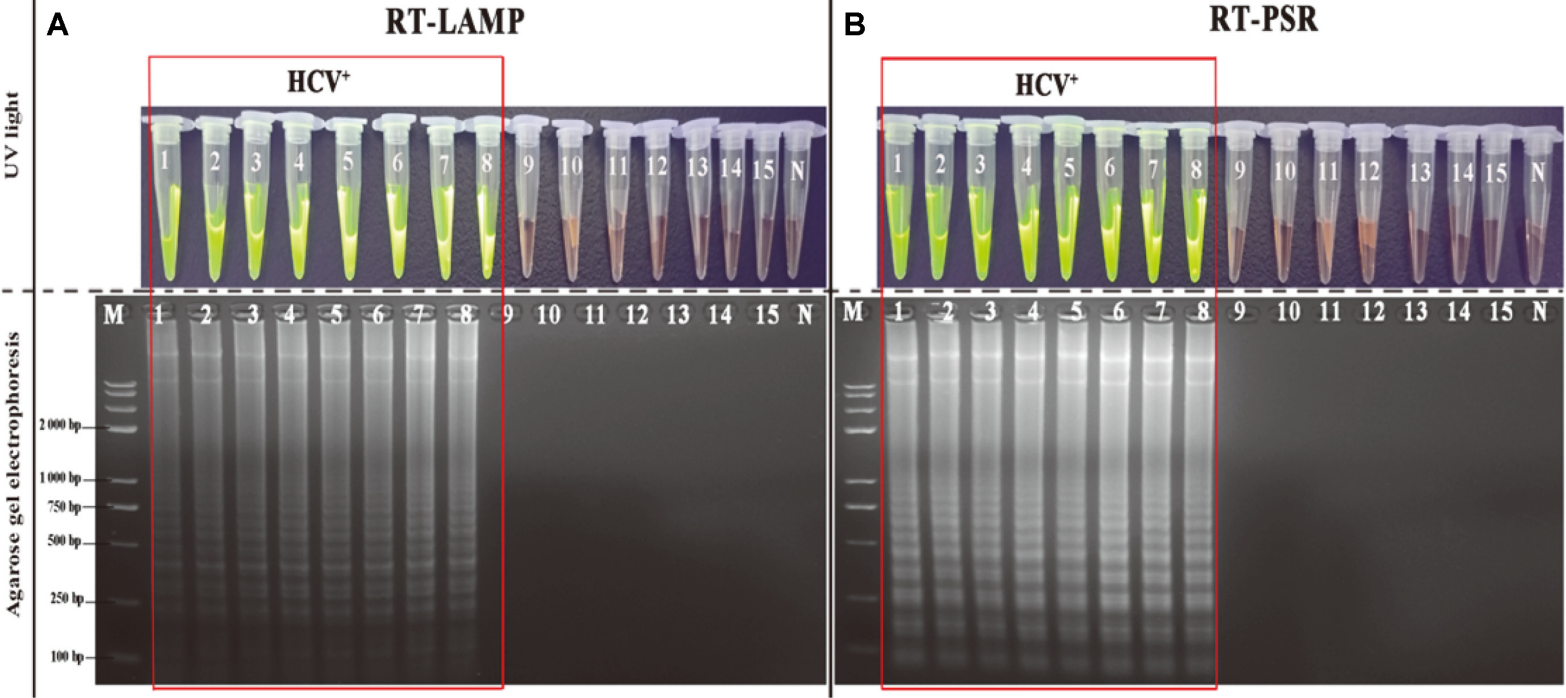
Effectiveness and specificity assessment of the RT-PSR assay for HCV detection. (**A** and **B**) Visualization of amplified DNA bands by RT-PSR and by RT-LAMP under UV light after agarose gel electrophoresis, respectively; M: Standard DNA marker. N: negative control. 1-15: HM674631, HEBEI L02836, AF034629, HK10U10197, STD11443, HK2U10198, HCV-15-1B, HCV- 36-2A, HB-8064, ATCC 13076, ATCC 50174, ATCC 25177, ATCC 25923, ATCC 70603 and ATCC 27519, respectively. (Detailed background of mentioned strains was shown in Table 1).

In order to evaluate the versatility and specificity of the RT-PSR assay, a panel of 15 relevant strains, including 6 synthetic HCV mimic strains, 2 HCV clinical isolates and 7 non-HCV purchased strains (Table 1) were examined by RT-PSR and RT-LAMP. The amplification products of all strains were visualized by closed isothermal amplification tube (IAT) under ultraviolet light after analysis in 1.8%agarose electrophoresis. The positive results with green fluorescence (dyes binding to double-stranded DNA) under the UV light were found only in the 8 HCV-positive strains and showed a ladder-like pattern in agarose electrophoresis, while no signals were observed in all 7 of non-HCV strains (Fig. 4). By comparison with the RT-LAMP assay, the RT-PSR results demonstrated a 100% specific and effective identification of HCV in the samples.

### Sensitivity of the RT-PSR Assay

**Fig. 5:**
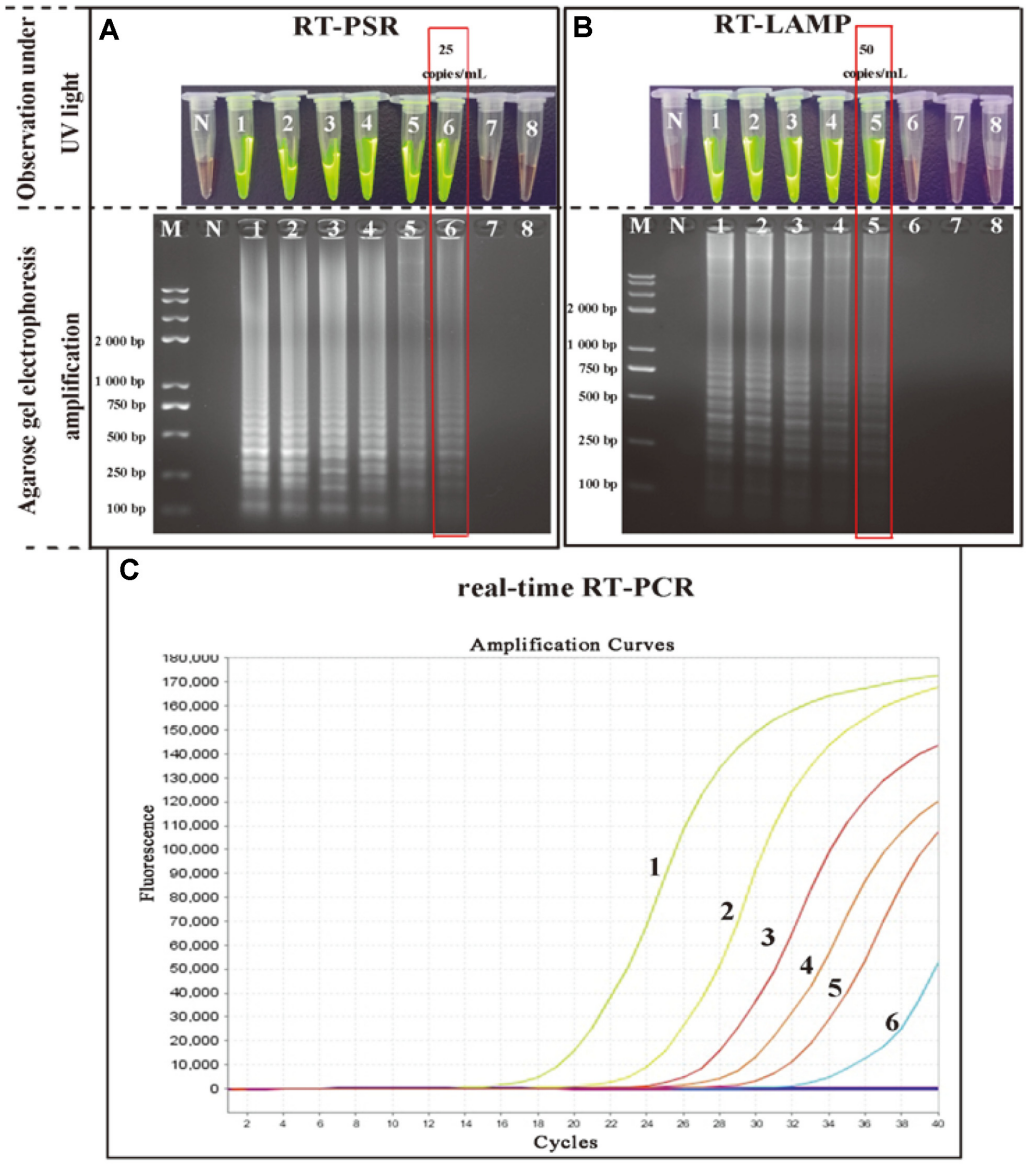
Evaluation of the sensitivity of RT-PSR, RT-LAMP and RT-PCR assays for HCV detection. (**A** and **B**) Visualization of amplified productions by RT-PSR and RT-LAMP under UV light, respectively. (**C**) Amplification results by real-time RT-PCR; M: Standard DNA marker; N: negative control. 1-8: 1 × 10^6^, 1 × 10^5^, 1 × 10^4^, 1 × 10^3^, 1 × 10^2^, 50, 25, 5 copies/ml.

To assess the limits of detection (LOD) of the RT-PSR assay compared with two conventional molecular diagnostic methods (RT-LAMP and real-time RT-PCR) in detection of HCV, known concentrations of HCV positive strains (1 × 10^6^ copies/mL) were serially diluted at 1 ×10^5^, 1 × 10^4^, 1 × 10^3^, 1 × 10^2^, 50, 25, 5 copies/mL then analyzed by RT-PSR, RT-LAMP and RT-PCR respectively. The results demonstrated that the detection limits of RT-PSR, RT-LAMP and real-time RT-PCR for the same cDNA sample were 25, 50, and 25 copies/ml respectively (Fig. 5). These data indicated that the LOD of the RT-PSR method is comparable with the real-time RT-PCR, but more sensitive than the RT-LAMP assay.

### Application of RT-PSR to Detect HCV in Clinical Samples

**Fig. 6:**
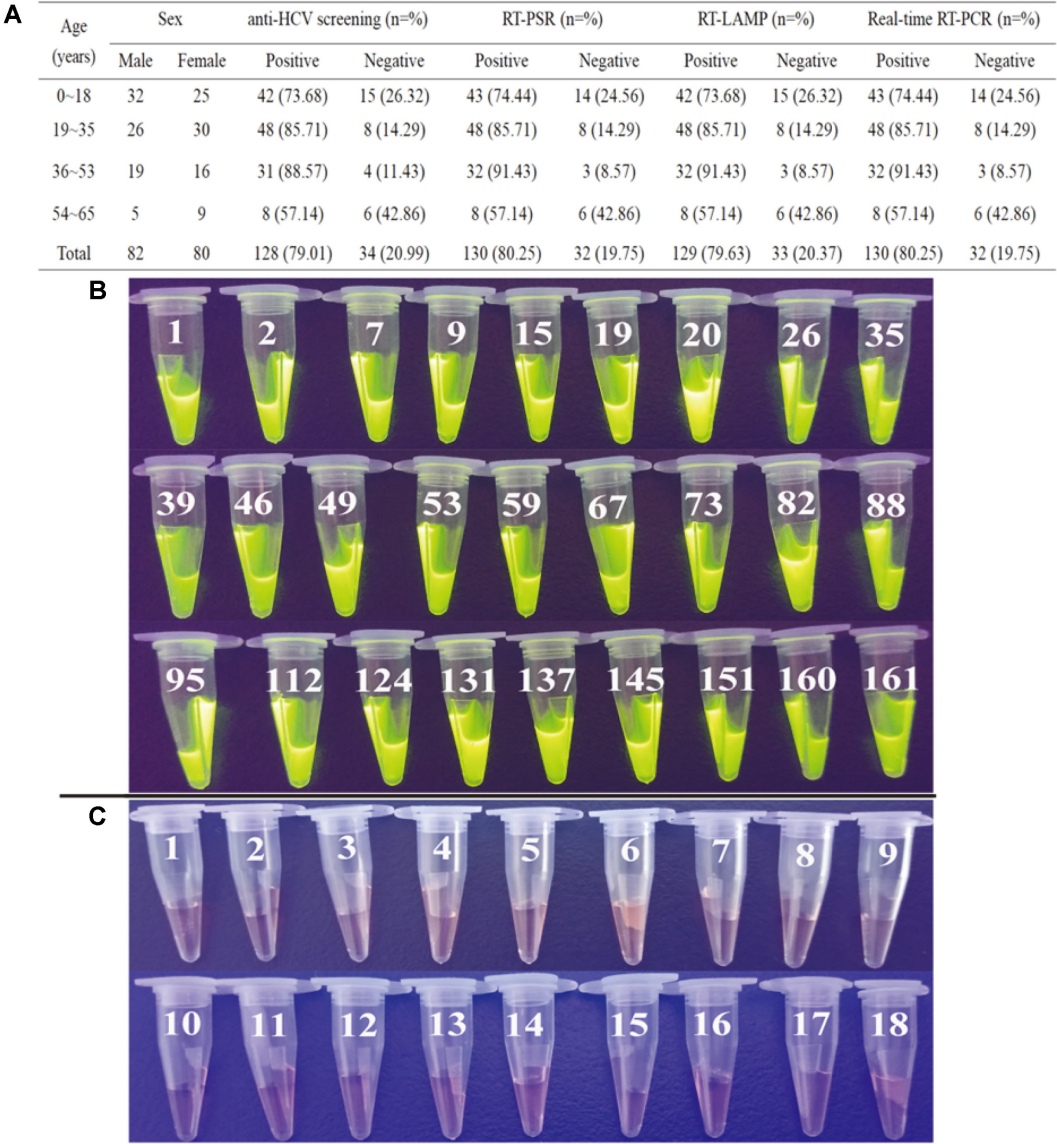
Evaluation of the sensitivity and effectiveness of RT-PSR assay for clinical blood samples. (**A**) Detailed detection results of 162 clinical blood samples who have suspected HCV infection by four independent assays (anti-HCV screening test, RT-PSR, RT-LAMP and real-time RT-PCR). (**B**) Partial results of RT-PSR assay in 162 clinical blood samples (under UV light). (**C**) Results of 18 healthy blood samples by RT-PSR assay (under UV light).

To better evaluate the effectiveness and specificity of the RT-PSR for detection of HCV, 180 blood samples collected from volunteer donors including 162 patients who have suspected HCV infection and 18 healthy controls were analyzed and compared to RT-LAMP, RT-PCR and the serological anti-HCV results as well. The results showed that, compared to serological screening which revealed 128 positive among 162 patient samples (the positive detection rate is 79.01%), the positive rates detected by RT-PSR, RT-LAMP, real-time RT-PCR are 80.25%, 79.63% and 80.25%respectively (Fig. 6). The data demonstrated that the sensitivity and effectiveness of RT-PSR for HCV detection in clinical samples are comparable to RT-PCR and are slightly better than RT-LAMP, which is consistent with the results of HCV-positive strains suggesting an effective potential alternative assay for existing assays.

## Discussion

Nearly 3% of the world’s population has been reported to be infected with HCV, which is a particularly serious threat to public health especially in developing countries [4]. The genotypes of HCV show different epidemic trends according to different geographical regions, which makes the prevention and treatment of hepatitis C disease more complex. Diagnosis of HCV in blood samples is an essential and critical step in providing better treatment and monitoring of patients. Therefore, a quick, low-cost, but more sensitive and effective assay is required. Currently, commercial diagnostic tests such as those based on serological screening including ELISA, recombinant immunoblot assay (RIBA) or nucleic acid test (NAT), are widely used and acceptable. However, the disadvantages largely limit their application especially in under-developed regions due to reasons such as the high cost, false positive results from non-specific cross reactions and the limitation of the availability of the equipment and so on [10]. The molecular-based diagnostic methods such RT-PCR and RT-LAMP solved some existing problems, but also have disadvantages such as expensive equipment and the lack of professional technicians, which restrict their clinical application and promotion.

The RT-PSR is a newly developed, PCR-based assay. The assay fulfills the WHO concept of affordable, sensitive, specific, user-friendly, rapid and robust, equipment-free and delivered (ASSURED) for disease diagnosis [25]. The RT-PSR assay in this study, by targeting the 5’UTR conserved region of the HCV gene, demonstrates advantages over labor-intensive methods that require professional training and multiple HCV detection diagnostic steps, which use only two primers similar to conventional PCR to avoid contaminations caused by using of multiple primers and the difficulty in primer design. In terms of optimization assay, the RT-PSR reactions were achieved at 63°C for 45 min, with 12 U *Bst*-DNA polymerase, 1.5 mM of dNTP, 1.2 M betaine and 4.0 mM of MgSO4, which are no different from the composition of conventional systems. Similar to other isothermal amplification methods, the RT-PSR assay enables users to visually monitor the success of amplification by active incorporation of the SYBR Green I dye, but opening the reaction tube will undoubtedly increase the risk of contamination caused by the large number of aerosols generated. The use of isothermal amplification tubes ensures that the amplification process is carried out in a closed device to reduce the risk of aerosol contamination and prevent false positive results. However, this method requires an increase in total reaction volume (≥30 µl) and should be mixed slowly after the end of the reaction to avoid bubbles generated by violent shaking. In addition, the development of a new type of universal detection device for preventing contamination is also feasible, and such a device could be based on colloidal gold immune-chromatography for the detection of HCV.

In this study, the diagnostic performance of RT-PSR assay was compared with two widely used assays, RT-LAMP and real-time RT-PCR. Our results showed that the RT-PSR has high specificity and versatility. It can identify any genotype of HCV^+^ without cross-reacting with each other and other non-specific strains, which is also consistent with the RT-LAMP assay. In terms of the sensitivity, the detection limit of the RT-PSR assay (≥25 copies/ml) is comparable with real-time RT-PCR assay for detection of HCV, but is two times higher than the RT-LAMP assay. To evaluate the feasibility of RT-PSR in the diagnosis of clinical blood samples, the results derived from serological anti-HCV screening were selected as reference. The RT-PSR test results indicated 80.25% (130/162) of positive detection, which is equal to the results detected by real-time RT-PCR, but slightly higher than the 79.63% positive detection rate when compared to RT-LAMP, indicating that RT-PSR is more sensitive in detection of HCV. The data we presented here demonstrated that the RT-PSR is a fast, effective and sensitive assay for the identification of HCV. This study will be very useful in the future either in clinical diagnosis or the epidemic study of the chronic hepatitis C.

In conclusion, we established and evaluated a novel RT-PSR assay for detection of HCV which has higher sensitivity than the previously established methods with encouraging results and provides a potential alternative assay for clinical assessment of HCV infection, especially in primary hospitals or laboratories.

## Figures and Tables

**Table 1:** Reference strains used in this study.

Species	Strain	Source	RT-PSR	RT-LAMP	Real-time RT-PCR
Hepatitis C virus 1a	HM674631	Synthesized	+	+	+
Hepatitis C virus 1b	HEBEI L02836	Synthesized	+	+	+
Hepatitis C virus 2a	AF034629	Synthesized	+	+	+
Hepatitis C virus 3a	HK10U10197	Synthesized	+	+	+
Hepatitis C virus 3b	STD11443	Synthesized	+	+	+
Hepatitis C virus 6a	HK2U10198	Synthesized	+	+	+
Hepatitis C virus	HCV-15-1B	Self-isolate	+	+	+
Hepatitis C virus	HCV-36-2A	Self-isolate	+	+	+
Hepatitis B virus	HB-8064	ATCC	－	－	－
*Salmonella*	ATCC 13076	ATCC	－	－	－
*Toxoplasma gondii*	ATCC 50174	ATCC	－	－	－
*Mycobacterium tuberculosis*	ATCC 25177	ATCC	－	－	－
*Staphylococcus* aureus	ATCC 25923	ATCC	－	－	－
*Klebsiella pneumoniae*	ATCC 70603	ATCC	－	－	－
*Vibrio* parahaemolyticus	ATCC 27519	ATCC	－	－	－

ATCC: American Type Culture Collection. Self-isolates were preserved in our lab. +: positive result. -: negative result.

**Table 2:** Primers of RT-PSR, RT-LAMP and real-time RT-PCR assays.

Name	Primer ID	Primers Sequence (5'-3')
H-PSR1	Ft1	gtcaaagcgatcccgccttac-TGGCGTTAGTATGAGTGTCGT
	Bt1	cattccgccctagcgaaactg-AAGCACCCTATCAGGCAGTA
H-PSR2	Ft2	gtcaaagcgatcccgccttac-GGATCAACCCGCTCAATG
	Bt2	cattccgccctagcgaaactg-AAGCACCCTATCAGGCAGT
H-PSR3	Ft3	gtcaaagcgatcccgccttac-GCGTTAGTATGAGTGTCGT
	Bt3	cattccgccctagcgaaactg-AGGCATTGAGCGGGTTG
RT-LAMP	FIP	GATCCAAGAAAGGACCCGGTTTTTCTGCGGAACCGGTGAGTAC
	BIP	CCTGGAGATTTGGGCGTGCTTTTAGTACCACAAGGCCTTTCGC
	F3	TCCCGGGAGAGCCATAGTG
	B3	CACTCGCAAGCACCCTATCAG
	Loop F	TCATCCTGGCAATTCCG
	Loop B	GCGAGACTGCTAGCCGAG

**Table 3:** Optimization of RT-PSR assay for detection of HCV by targeting the 5’ UTR region.

Conditions	The test interval	Difference	Good yield	Final selected^a^
Temperature	61 ~ 71 ℃	1 ℃	62 ~ 64 ℃	63 ℃
MgSO4	1.0 ~ 8.0 mM	1.0 mM	4 ~ 6 mM	4 mM
dNTPs	1.0 ~ 4.0 mM	0.5 mM	1.5 ~ 3.0 mM	1.5 mM
Betaine	0.6 ~ 1.4 M	0.1 M	0.8 ~ 1.2 M	1.2 M
*Bst* DNA polymerase	6 ~ 18 U	2 U	12 ~ 18 U	12 U
Time	30 ~ 120 min	15 min	30 ~ 60 min	45 min
